# Perioperative Care of a Four-Year-Old Child With Teebi Hypertelorism Syndrome: A Rare Craniofacial Disorder

**DOI:** 10.14740/jmc5268

**Published:** 2026-03-04

**Authors:** Eslam Abdelhady, Joseph D. Tobias

**Affiliations:** a Department of Anesthesiology and Pain Medicine, Nationwide Children’s Hospital, Columbus, OH, USA; b Department of Anesthesiology and Pain Medicine, The Ohio State University College of Medicine, Columbus, OH, USA

**Keywords:** Teebi hypertelorism syndrome, Craniofacial syndrome, Facial cleft

## Abstract

Teebi hypertelorism syndrome (THS) is a rare, autosomal dominant craniofacial malformation disorder, characterized by orbital hypertelorism and characteristic craniofacial features, including a prominent forehead, wide arched eyebrows, short broad nasal root and tip, a thin upper lip, and a small chin. In addition to the characteristic phenotypic traits, systemic malformations may involve the limbs, central nervous system, urogenital tract, uterus, umbilicus (omphalocele), or cardiac system. Most cases can now be attributed to pathogenic variants in the *SPECC1L* gene on chromosome 22q11.23, leading to what is known as SPECC1L-related hypertelorism syndrome, which is associated with disruption of a cytoskeletal cross-linking protein. This protein is crucial for normal craniofacial morphogenesis during neural crest cell migration and midline facial formation. To date, there are no previous reports of anesthetic care in a patient with THS. We present a 4-year-old child with THS who presented for anesthetic care during thoracic laminectomy and excision of an intradural mass. We explore the history and clinical presentation of the disorder, outline end-organ involvement, and discuss perioperative concerns.

## Introduction

Teebi hypertelorism syndrome (THS) is a rare craniofacial malformation disorder, characterized by significant orbital hypertelorism, with a prominent forehead, wide arched eyebrows, and a short broad nasal root and tip. Airway involvement may include a thin upper lip, micrognathia, cleft palate, and limited mouth opening. Beyond these craniofacial traits, some individuals exhibit systemic findings involving the limbs, urogenital tract, umbilicus, or cardiac anomalies [1]. The condition was first described in 1987 by Ahmad S. Teebi, who reported the disorder in an Arab family showing a consistent facial pattern across several generations, establishing THS as an autosomal dominant disorder [2]. Both genders were noted to be equally affected, supporting this pattern of inheritance. Recently, molecular genetics studies have highlighted the underlying heterogeneity of THS. Most cases can now be attributed to pathogenic variants in the *SPECC1L* gene on chromosome 22q11.23, leading to what is known as SPECC1L-related hypertelorism syndrome [3, 4].

The *SPECC1L* gene encodes a cytoskeletal cross-linking protein crucial for normal craniofacial morphogenesis, involved in neural crest cell migration, midline facial formation, and palate development [5, 6]. Disruption involving this pathway leads to defects in cytoskeletal organization and cell adhesion, resulting in the familiar midline malformations seen in THS, including hypertelorism, cleft lip/palate, and frontonasal dysplasia [4]. More recently, variants in *CDH11*, located on chromosome 16q21, have been linked to a Teebi-like phenotype. As *CDH11* encodes cadherin-11, a protein important for cell–cell adhesion, these findings highlight the locus heterogeneity of THS [7].

THS remains exceedingly rare with an estimated incidence of less than 1:1,000,000 with fewer than 1,000 affected individuals in the United States [1, 2]. To date, there are no previous reports regarding anesthetic care in these patients. We present a 4-year-old child with THS who presented for anesthetic care during thoracic laminectomy and excision of an intradural mass. We explore the history and clinical presentation of the disorder, outline end-organ involvement, and discuss perioperative concerns.

## Case Report

Review of this case and presentation in this format followed the guidelines of the Institutional Review Board of Nationwide Children’s Hospital. Written consent was obtained for anesthetic care as well as the use of deidentified patient information for education and/or publication.

A 4-year-old boy weighing 25.2 kg with THS, presented to the Neurosurgery Clinic with complaints of leg and back pain, occurring one to two times per week, a past history of delayed walking at 2 years of age, and frequent falls. On examination, the patient was in no acute distress, and his parents denied any other symptoms. Magnetic resonance imaging (MRI) revealed findings consistent with a dermoid or inclusion cyst in the thoracic region, stable postoperative changes of the previous thoracic myelocele repair, stable small fatty filum, and no evidence of cord tethering. Past medical history included being large for gestational age, cerebral ventriculomegaly, spina bifida, and a high-risk social situation, with a history of substance abuse in his maternal grandfather, natural father, and natural mother. He was adopted and at 7 months of age, was diagnosed with THS, confirmed by the presence of *SPECC1L* gene mutation. Prior surgeries included an uneventful previous procedure to close the thoracic myelomeningocele and multiple head, cervical, thoracic, and lumbar MRIs without notable anesthetic concerns.

At the time of the preoperative evaluation, the patient weighed 25.5 kg. Preoperative vital signs included a heart rate of 85 beats/min, blood pressure 121/77 mm Hg, and room air oxygen saturation of 100%. His airway assessment revealed a thyromental distance of two fingerbreadths and a Mallampati II examination despite the presence of micrognathia. There was normal range of motion of the neck. Cardiac and respiratory examinations were unremarkable. He was assigned an American Society of Anesthesiologists (ASA) physical status III. Preoperative laboratory evaluation included a complete blood count, which showed mild anemia (hemoglobin 10.5 g/dL), and a comprehensive metabolic panel, which was within normal limits. Outpatient medications at the time of surgery included oral cetirizine once daily, lactulose and polyethylene glycol to prevent constipation, and oral acetaminophen as needed. There were no drug allergies. The patient was held *nil per os* for 8 h and transported to the operating room where routine ASA’ monitors were placed. Anesthesia was induced by the inhalation of incremental concentrations of sevoflurane in oxygen and nitrous oxide. Following anesthetic induction, a peripheral intravenous (IV) cannula was placed, and midazolam (0.1 mg/kg) and fentanyl (4 µg/kg) were administered. Following the demonstration of adequate bag-valve-mask ventilation, rocuronium (0.8 mg/kg) was administered to facilitate endotracheal intubation. The patient’s trachea was intubated orally with a 5.0-mm cuffed endotracheal tube, using direct laryngoscopy with a grade 1 glottic view. Following endotracheal intubation, a second peripheral IV cannula and a left radial arterial cannula were placed using ultrasound guidance. A urethral catheter was placed to monitor core body temperature and urine output. To facilitate neurophysiologic monitoring during tumor resection, maintenance anesthesia included methadone and a remifentanil infusion (0.1–0.3 µg/kg/min), propofol (100–250 µg/kg/min) titrated to maintain the bispectral index at 50–60, and lidocaine (1 mg/kg/h). No additional neuromuscular blocking agents (NMBAs) were administered. The patient was positioned prone on a Jackson table. All pressure points were identified and appropriately padded. Normothermia was maintained by control of the room temperature and a forced air warming blanket. Augmentation of coagulation function to limit the need for allogeneic blood products was achieved by the administration of tranexamic acid (bolus dose of 50 mg/kg followed by an infusion at 5 mg/kg/h). Prophylaxis to prevent surgical site infection was achieved using cefazolin (50 mg/kg every 3 h). The intraoperative course was uneventful with a total operating room time of 6.5 h. No blood products were administered intraoperatively. Two transient episodes of hypotension were treated with phenylephrine (1 µg/kg). Intraoperative fluids included 820 mL of Normosol®-R. Estimated blood loss was 365 mL. Hydromorphone (0.02 mg/kg) and acetaminophen (15 mg/kg) were administered for postoperative pain control. Prophylaxis to prevent postoperative nausea and vomiting included dexamethasone (0.2 mg/kg) and ondansetron (0.15 mg/kg). At the completion of the surgical procedure, the anesthetic infusions were discontinued, the patient was turned supine, and his trachea was extubated when he was awake. He was transferred to the post-anesthesia care unit (PACU) and then to the pediatric intensive care unit (PICU) for close monitoring of his cardiorespiratory function. During the initial postoperative period, mild oxygen desaturation on room air (saturation 80–85%) required the administration of supplemental oxygen (2 L via nasal cannula). The remainder of the postoperative course was unremarkable. The patient was weaned to room air by the next morning. Postoperative analgesia was provided by fixed interval dosing of acetaminophen every 6 h and morphine delivered by nurse-controlled analgesia. On postoperative day (POD) 1, an MRI of the thoracic spine revealed complete excision of the thoracic mass. Anesthesia was provided for the MRI with sevoflurane in air and oxygen delivered via a laryngeal mask airway (LMA), which was tolerated without incident. The patient was then transferred back to the PICU. The remainder of his postoperative course was unremarkable, and he was discharged home on POD 4.

## Discussion

Given the potential for multiple associated anomalies and end-organ involvement with THS, anesthetic care may be required during radiologic imaging or surgical interventions. To date, there are no previously published reports outlining the specific anesthetic care of a patient with THS. In general, anesthetic care should begin with a thorough preoperative evaluation examining the patient’s past medical history, current medication regimen, and previous surgical care. The preoperative examination should identify specific end-organ involvement related to the primary underlying condition as well as unrelated issues, both acute and chronic, which may impact perioperative care. With THS, in addition to the characteristic craniofacial features (prominent forehead, broad nasal bridge, thin upper lip, and small chin), end-organ and congenital anomalies may include the limbs, umbilicus (omphalocele), central nervous system, urogenital tract, gynecologic tract, or cardiac system. Anesthetic concerns regarding Teebi syndrome end-organ and congenital anomalies are summarized in Table 1. The extent of organ involvement often differs considerably among affected patients. This holds true even for family members who share the same genetic mutation [6, 7].

**Table 1:** End-Organ Involvement and Perioperative Concerns in Patients With Teebi Hypertelorism Syndrome

System	Clinical features	Anesthetic concerns
Airway and craniofacial	Characteristic facial features including midface hypoplasia, micrognathia, short and broad nasal bridge	Difficult mask ventilation, as midface hypoplasia and broad nasal bridge may prevent an effective mask seal
	Oropharyngeal involvement including a small oral cavity or cleft lip/palate	Difficult endotracheal intubation due to micrognathia and small oral cavity, which may impair alignment of oral-pharyngeal-laryngeal axes
		Risk of upper airway obstruction during sedation with a native airway or following recovery from anesthesia
Cardiovascular	Congenital heart disease (patent ductus arteriosus, atrial or ventricular septal defects)	Preoperative echocardiography is recommended.
	Valvular involvement (aortic stenosis)	Potential for sensitivity to myocardial depressants
	Root dilatation	Maintenance of systemic venous return is critical in shunting lesions.
		Potential for paradoxical embolism with septal defects and intracardiac shunts
CNS	CNS malformations and structural lesions including ventriculomegaly, agenesis of the corpus callosum, spinal dysraphism (spina bifida, tethered cord)	Intracranial pressure alterations may require consideration of the impact of anesthetic agents or intraoperative maneuvers that impact intracranial pressure.
	Spina bifida/myelocele or tethered cord (as noted in case discussion)	Cognitive impairment may impact cooperation and communication during perioperative care.
	Functional involvement including developmental delay, intellectual disability	
Musculoskeletal and extremity involvement	Limb anomalies including small hands/feet, interdigital webbing, clinodactyly	Vascular access including placement of peripheral IV and arterial cannula may be difficult. Ultrasound guidance is recommended.
		Attention to positioning is required with padding of pressure points and protection of neural repair sites (spina bifida repair) during long procedures.
Genito-urinary tract	Cryptorchidism (undescended testes) and shawl scrotum in males	Preoperative assessment of renal function based on past history with imaging (ultrasound) as needed
	Bicornuate uterus in females	
Gastrointestinal tract	Umbilical hernia and/or omphalocele	Preoperative imaging based on physical assessment and history
	Congenital diaphragmatic hernias or tracheoesophageal	
	Absent gallbladder	
	Anorectal malformations	

CNS: central nervous system; IV: intravenous.

Patients with known genetic syndromes, especially those with craniofacial involvement, may pose a variety of challenges to the anesthesia provider, including the potential for difficulties with airway management, bag-valve-mask ventilation or endotracheal intubation [8–10]. Craniofacial abnormalities, including midface hypoplasia, a short and broad nasal bridge, and micrognathia, represent key characteristics of THS. These features elevate the risk of difficult bag-valve-mask ventilation due to the inability to achieve an effective seal and difficult endotracheal intubation related to mid-face hypoplasia, small oral cavity, micrognathia, and limited jaw movement [11]. A smaller oral cavity may impact direct laryngoscopy, while limited jaw movement with micrognathia impairs the alignment of oral, pharyngeal, and laryngeal axes [12]. Additional concerns regarding airway management include the association of SPECC1L-related disorders, with a range of cleft and mid-face anomalies ranging from isolated cleft palate to frontonasal dysplasia [3]. Anatomic distortion in the nasopharynx or oropharynx can obscure the glottic view during direct laryngoscopy. These factors can also place the patient at risk for upper airway obstruction during sedation with a native airway or following recovery from general anesthesia when the residual effects of anesthetic agents may impact upper airway tone and control. In our patient, spontaneous ventilation was maintained during the inhalation induction of anesthesia with sevoflurane in oxygen while the administration of a NMBA, rocuronium, proceeded only after effective bag-valve-mask ventilation had been demonstrated. Endotracheal intubation was uneventful despite mild micrognathia. When caring for such patients, the equipment to deal with the difficult airway, including indirect video-laryngoscopy, should be readily available [12, 13].

Associated anomalies may also affect the limbs, central nervous system, urogenital tract, uterus, umbilicus (omphalocele), or cardiac system in patients with THS. Associated involvement of the cardiac system includes congenital heart disease (patent ductus arteriosus, atrial or ventricular septal defects), valvular involvement (aortic stenosis) or root dilatation [4]. A single anecdotal report notes the occurrence of third-degree atrioventricular block in a patient with THS who required placement of a pacemaker [14], but the association of conduction defects with THS remains anecdotal. Given these concerns, a preoperative echocardiogram is suggested to rule out associated CHD along with an electrocardiogram when conduction defects are suspected based on history or clinical findings.

The association of central nervous system involvement with THS is characterized by significant phenotypic variability. Anatomic involvement may include ventriculomegaly, agenesis of the corpus callosum, or spinal/vertebral defects (spina bifida and thoracic myelocele were observed in our patient) [15]. Functionally, variants in SPECC1L tend to link with intellectual disability, and less consistently with seizure disorders, which are more specifically associated with anatomical/structural involvement [3, 4]. Preoperative assessment and perioperative impact vary based on the specific structural involvement, cognitive impairment, and associated presence of a seizure disorder.

Limb abnormalities in THS are generally mild and variable. Reported features include small hands and feet, broad hands, webbing between fingers, and clinodactyly of the fifth finger [3, 16]. Abnormal limb anatomy may impact peripheral venous and arterial cannulation. Ultrasound guidance, as used in our case, is recommended to facilitate vascular access. Additionally, patients with skeletal asymmetry or contractures may require attention to padding and positioning during prolonged procedures in the prone position [17].

In addition to general considerations for postoperative care, patients with THS may require specialized care to mitigate the risks of upper airway obstruction postoperatively due to associated craniofacial and the residual effects of anesthetic agents [18–20]. In our patient, we chose to use short-acting anesthetic agents (remifentanil) to limit the impact of the residual effects of anesthetic agents on postoperative respiratory function, along with continuous postoperative monitoring of respiratory function in the PICU setting. Additionally, the use of an intermediate-acting NMBA (rocuronium), reversal of residual neuromuscular blockade with sugammadex, and the use of quantitative neuromuscular monitoring are recommended to guide dosing of NMBAs and facilitate early tracheal extubation.

### Learning points

In summary, we present the successful anesthetic management of a patient with THS. THS is a rare, autosomal dominant craniofacial malformation disorder, characterized by characteristic facial features, including mid-line abnormalities such as cleft lip/palate and orbital hypertelorism, and is associated with systemic malformations involving the limbs, central nervous system, urogenital tract, uterus, umbilicus (omphalocele), or cardiac system. This sequence of abnormalities has been linked to pathogenic variants in the *SPECC1L* gene on chromosome 22. The *SPECC1L* gene is responsible for a cross-linking protein involved in normal craniofacial development, regulating neural crest cell migration, midline facial formation, and palate development. A thorough preoperative evaluation is required to identify end-organ involvement and plan for perioperative care. Of primary importance is the potential for craniofacial features that may impact airway management and associated CHD. Craniofacial abnormalities may increase the risk of difficult bag-valve-mask ventilation and endotracheal intubation. Neurologic involvement may include both structural and functional involvement of the central nervous system. Ultrasound guidance may be useful during vascular access due to related limb anomalies. In the postoperative setting, close respiratory monitoring and the use of short-acting anesthetic agents can help minimize the risk of airway obstruction and facilitate recovery from anesthetic care.

## Data Availability

Any inquiries regarding supporting data availability of this study should be directed to the corresponding author.
